# Risk of Malignancy in Long-Standing Goiters: A Retrospective Study at Khartoum Teaching Hospital, Sudan

**DOI:** 10.7759/cureus.86931

**Published:** 2025-06-28

**Authors:** Mohamed A Ali, Mohamed ElMakki Ahmed

**Affiliations:** 1 Surgery, Sudan Medical Specialization Board, Khartoum, SDN; 2 Surgery, Khartoum Teaching Hospital, Khartoum, SDN

**Keywords:** incidental carcinoma, long-standing goiter, retrospective observational study, thyroid cancer, thyroidectomy, thyroid malignancy

## Abstract

Background: Goiter is a common condition in Sudan, often presenting with a wide range of clinical features. While the risk of malignancy in long-standing goiters is generally considered low, it remains a clinical concern, especially with a prolonged disease duration. Surgical intervention should be considered in managing goiters of prolonged duration to mitigate this risk.

Aims: This study aims to evaluate the risk of malignancy and identify the subtypes of malignant transformation in patients with long-standing goiters undergoing thyroidectomy at Khartoum Teaching Hospital, Sudan.

Methods: This retrospective descriptive study was conducted at Khartoum Teaching Hospital, a major tertiary care center in Sudan, over 18 months from March 2017 to September 2018. A total of 160 patients aged 18 years and above with long-standing goiters scheduled for thyroidectomy were included. Data were collected from patient medical records, capturing demographic information, clinical characteristics, and histopathological findings, and compiled directly into a spreadsheet. Preoperative, intraoperative, and postoperative assessments included clinical examinations and thyroid function tests. Histopathological analysis of thyroid specimens determined the presence and type of malignancy. Statistical analysis was performed using IBM SPSS Statistics for Windows, Version 25 (Released 2017; IBM Corp., Armonk, New York, United States), with chi-square (χ²) tests applied to assess associations, and a p-value of <0.05 was considered statistically significant.

Results: The mean age of participants was 52.5 ± 14 years, with a male-to-female ratio of 1:4.5. Pressure symptoms were reported in 151 patients (94.4%), and 11 patients suffered from recurrent laryngeal nerve injury and five patients were found to have metastasis at the time of surgery. Histopathological examination revealed malignancy in 54 patients (32.5%), with follicular carcinoma being the most common subtype. Among the malignant cases, 44 were clinically suspected, while 10 (9%) were incidentally discovered. A significant association was observed between clinical diagnosis and histopathological confirmation of malignancy (P = 0.001).

Conclusion: This study found a significant rate of malignancy among patients with long-standing goiters undergoing thyroidectomy at Khartoum Teaching Hospital, with follicular carcinoma being the most common subtype. Many cases were incidentally discovered, highlighting the need for thorough preoperative evaluation and patient counseling in regions with endemic goiter.

## Introduction

Goiter, characterized by the enlargement of the thyroid gland, remains a significant public health concern in Sudan. Despite the implementation of iodine supplementation programs since the 1970s, including universal salt iodization, the prevalence of goiter remains high. For instance, a study conducted in South Kordofan reported a goiter prevalence of 42.8% among children aged 6-12 years, which highlights the ongoing challenges in iodine deficiency disorders in the region [1]. In Shendi, a River Nile State, the overall prevalence of goiter was 18%, with the highest prevalence in Hajer-Alasal at 5.3% and the lowest in Shendi town at 3.6%. Goiter was more common among women than men at a ratio of 3:1, and it was particularly prevalent in the age group of 31 to 45 years [2]. Additionally, in another study conducted in rural South Sudan, iodine deficiency was found to be a major cause of goiter, with 25% of goiter patients showing moderate to severe iodine deficiency, where the majority of the goiter patients were female [3].

Iodine deficiency is a primary etiological factor for goiter development [4]. However, other contributors such as vitamin A deficiency, protein-energy malnutrition, and the consumption of goitrogenic substances like thiocyanate found in pearl millet, a staple in Sudanese diets, also play roles in the endemicity of goiter [5]. Chronic iodine deficiency increases oxidative stress and may damage DNA, potentially facilitating the transformation of benign nodular goiters into malignant forms.

Thyroid malignancies are notably more prevalent in regions with endemic goiter. Studies have indicated that the incidence of thyroid carcinoma in multinodular goiters ranges from 3% to 31% [6]. The predominant types of thyroid cancers in these settings are follicular and papillary carcinomas, both originating from follicular cells. These malignancies can metastasize to lymph nodes, bones, and lungs, sometimes presenting initially through these secondary sites [7].

Khartoum Teaching Hospital, which is a major tertiary care center in Sudan, provides specialized services in endocrinology and surgery, serving a diverse patient population from both urban and rural areas. Given the high prevalence of long-standing goiters and the associated risk of malignancy, this study aims to evaluate the risk of malignancy and identify the subtypes of malignant transformation in patients with long-standing goiters undergoing thyroidectomy at Khartoum Teaching Hospital.

## Materials and methods

This is a retrospective descriptive study, conducted in Khartoum Teaching Hospital, one of the major tertiary care hospitals in Sudan, known for its specialized services in endocrinology and surgery. The hospital serves a diverse patient population from urban and rural areas. The study collected information from an 18-month time frame, from March 2017 to September 2018.

The study population included all adult patients of both genders (aged 18 years and above) diagnosed with long-standing goiters (≥10 years) who presented to Khartoum Teaching Hospital during the study period from March 2017 to September 2018. Eligibility for inclusion required that patients had received medical treatment and undergone thyroidectomy within this timeframe. A total population (convenience sampling) approach was used, in which all eligible patients meeting the criteria were included; therefore, a sample size calculation was not required.

Data was collected using a thorough review of patient medical records. The review captured demographic information (age, gender), clinical characteristics (symptoms of goiter, co-morbidities, recurrence of goiter, and duration of goiter), and medical history, including diagnosis and types of malignancies. Preoperative evaluation included clinical examination and thyroid function tests (TSH, T3, T4). Intraoperative details such as the type of thyroidectomy performed, surgery duration, and any complications were recorded. Postoperative follow-up data collected involved histopathological analysis of thyroid specimens to detect malignancy and assess surgical complications, such as recurrent laryngeal nerve (RLN) injury and hypocalcemia.

Ethical approval for the study was obtained from the Ethical Committee of the Sudan Medical Specialized Board under approval number EA254000322, dated 3rd April 2025. Further permissions were secured from Khartoum Teaching Hospital for data collection and access to patient records. To maintain confidentiality, patient identifiers such as names, addresses, and tribal affiliations were omitted from the data collection. All collected information was securely stored and used exclusively for research purposes.

Data analysis was conducted using IBM SPSS Statistics for Windows, Version 25 (Released 2017; IBM Corp., Armonk, New York, United States). Age was presented as mean ± standard deviation (SD), while categorical variables, such as histopathological diagnosis and clinical features, were summarized as frequencies and percentages (n [%]). The chi-square (χ²) test was used to examine associations between categorical variables. A p-value of <0.05 was considered statistically significant.

## Results

A total of 160 patients with long-standing goiters (≥10 years) were included in this study. Patients were referred for surgery due to the development of pressure symptoms or because of the large size of the goiter. The patients’ ages ranged from 19 to 81 years, with a mean age of 52 ± 14 years. Table 1 summarizes the characteristics of the 160 patients included in the study to evaluate the risk of malignancy in long-standing goiters and to identify subtypes of malignant transformation. The age of participants ranged from 19 to 81 years, with a mean age of 52.5 ± 14 years. The cohort comprised 28 male patients (17.5%) and 132 female patients (82.5%), with a male-to-female ratio of 1:4.5. Regarding clinical presentations, pressure symptoms were reported in 151 patients (94.4%), whereas nine patients (5.6%) did not exhibit any pressure-related symptoms (Table 1).

**Table 1:** Patient Characteristics

Characteristics	N (%)
Total Participants	160
Male	28 (17.5%)
Female	132 (82.5%)
Age (Years)	19–81 (Mean ± SD: 52.5 ± 14)
Pressure Symptoms	
Present	151 (94.4%)
Absent	9 (5.6%)

For diagnostic assessment, thoracic inlet X-ray was performed in 119 patients (74.4%), and CT scans were conducted in 41 patients (25.6%). Additionally, recurrent goiter was noted in 28 patients (17.5%), while 132 patients (82.5%) showed no signs of recurrent goiter.

**Figure 1:**
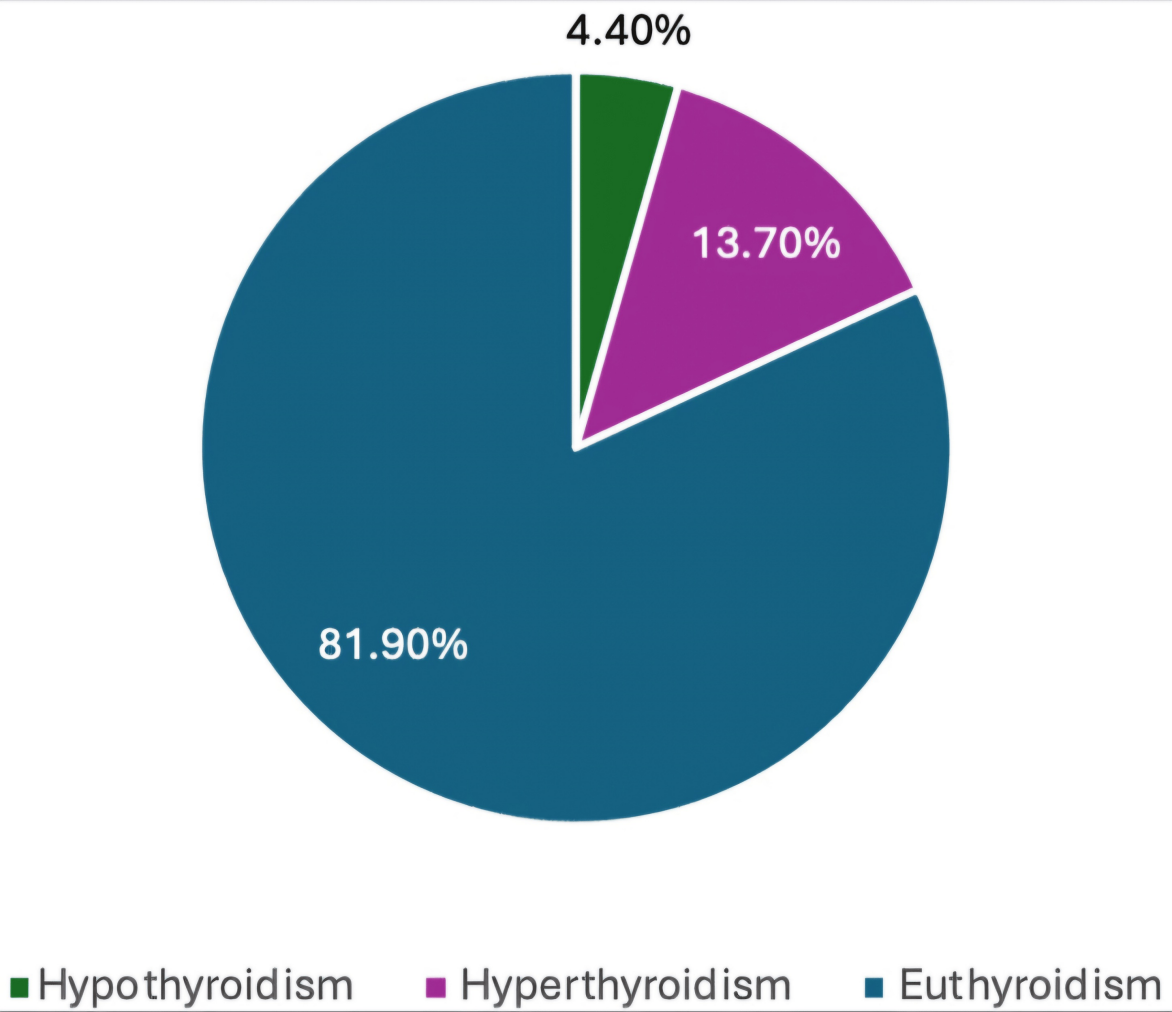
Thyroid Status Distribution Among Patients with Long-Standing Goiters

The distribution of thyroid status among patients with long-standing goiters is presented in Figure 1. The majority of patients were euthyroid (131; 81.9%), followed by hyperthyroid (22; 13.7%) and hypothyroid (7; 4.4%). This distribution indicates that most individuals with long-standing goiters maintain normal thyroid function, with a smaller proportion exhibiting hyperthyroidism or hypothyroidism.

**Figure 2:**
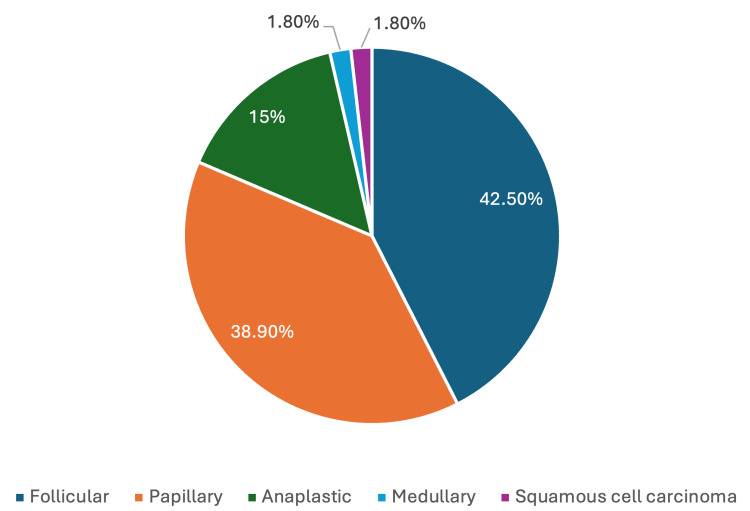
Distribution of Thyroid Malignancies in Long-Standing Goiters (N = 54)

Histopathological examination revealed that 106 patients (67.5%) had benign goiters, while 54 patients (32.5%) were diagnosed with malignant findings. Among the malignant cases, 44 patients were clinically suspected of malignancy and later confirmed by histopathological examination, while 10 cases (9%) were incidentally discovered in patients initially diagnosed with benign goiters. The distribution of thyroid malignancy subtypes among the 54 malignant cases is illustrated in Figure 2.

Table 2 summarizes the distribution of malignancy types among patients with long-standing goiters according to gender, clinical diagnosis, hypocalcemia status, disease duration, recurrence, and thyroid functional status. A significant association was observed between gender, clinical diagnosis, and disease duration with malignancy subtypes (P < 0.05). Follicular carcinoma was more prevalent in female patients, in patients clinically diagnosed as malignant, and in those with a disease duration of less than 20 years. In contrast, there were no significant associations between hypocalcemia, recurrence, or thyroid status and specific malignancy types (P > 0.05). 

**Table 2:** Distribution of the Patients with Long-Standing Goiters According to Factors Associated with Types of Malignancies * Significant P-value < 0.05, Chi-square test

Factors			Types of Malignancy						
			Follicular	Papillary	Medullary	Anaplastic	SCC	Chi-square value	P-value
Gender	Male		3	11	0	1	0	13.2	0.002*
	Female		20	10	1	7	1		
Clinically	Benign		5	6	0	0	1	15.6	0.001*
	Malignant		18	15	1	8	0		
Hypocalcemia	Yes		3	1	0	2	0	4.2	0.363
	No		20	20	1	6	1		
Duration	<20		21	19	0	8	0	16.8	0.001*
	>20		2	2	1	0	1		
Recurrent Goiter	Yes		5	3	1	1	0	3.9	0.356
	No		18	18	0	7	1		
Status	Hypothyroidism		1	0	0	1	0	3.6	0.398
	Hyperthyroidism		2	3	1	0	0		
	Euthyroidism		20	18	0	7	1		

Table 3 presents the correlation between clinical diagnosis and histopathological findings in patients with long-standing goiters. Among those clinically diagnosed as benign (n=116), 11 patients (9.5%) were found to have malignant pathology upon histopathological examination. In contrast, out of those clinically suspected as malignant (n=44), 41 patients (93.2%) were histopathologically confirmed as malignant, while three patients (6.8%) were found to have benign pathology. The association between clinical diagnosis and histopathological confirmation was statistically significant (P = 0.001), indicating a strong correlation between clinical suspicion and actual malignancy outcomes. 

**Table 3:** Distribution of the Patients with Long-Standing Goiters According to Correlation between Clinical and Histopathological Findings

Clinical	Pathology			
	Benign	Malignant	Chi-square value	P-value
Benign	105	11	102.6	0.001*
Malignant	3	41		
* Significant P-value < 0.05, Chi-square test				

Metastasis was identified in 11 patients (6.9%). Additionally, postoperative complications included RLN injuries in five patients (3.1%) and hypocalcemia in 12 patients (7.5%). Among those with hypocalcemia, 11 patients (6.8%) experienced transient hypocalcemia, while one patient (0.6%) was diagnosed with permanent hypocalcemia (Table 4).

**Table 4:** Incidence of Metastasis and Postoperative Complications

Condition	Number of Patients (%)
Metastasis	11 (6.9%)
Recurrent Laryngeal Nerve (RLN) Injury	5 (3.1%)
Hypocalcemia (Total)	12 (7.5%)
Transient Hypocalcemia	11 (6.8%)
Permanent Hypocalcemia	1 (0.6%)

## Discussion

This study evaluated the risk of malignancy and identified subtypes of malignant transformation in patients with long-standing goiters at Khartoum Teaching Hospital. The findings revealed a notable prevalence of malignancy, with 54 cases (32.5%) among patients undergoing thyroidectomy, and follicular carcinoma was identified as the most common histopathological subtype. Additionally, a significant association was observed between malignancy and certain demographic and clinical characteristics, including female gender, clinical diagnosis, and disease duration. These results highlight the persistent risk of thyroid cancer in regions with endemic goiter, despite longstanding iodine supplementation programs.

The age distribution in this study ranged from 19 to 81 years, with a mean age of 52.5 ± 14 years, indicating that goiter predominantly affects middle-aged and elderly individuals. This trend is consistent with previous findings that reported the prevalence of thyroid nodules, often associated with or contributing to goiter, increases significantly with age, particularly among women and individuals with central obesity [8]. Among patients with multinodular goiter (MNG), the mean age was reported to be 56.4 years, and the prevalence of incidental thyroid cancer was notably high at 31.7%, with papillary thyroid carcinoma representing 89.4% of these cases [6]. However, younger age and male sex were identified as independent risk factors for incidental thyroid malignancy in patients with MNG [6].

The marked predominance of 28 male patients (17.5%) and 132 female patients (82.5%) aligns with global and regional data, which show that women are more commonly affected by thyroid disorders. Estrogen receptor expression in thyroid tissue and autoimmune susceptibility are hypothesized to underlie this gender disparity. This observation is further supported by a study that analyzed 712 patients with multinodular goiter and found that 87.5% of the cases were female. However, interestingly, gender was not identified as a statistically significant risk factor for malignancy (p = 0.169) [9]. While thyroid disorders are more prevalent in women, the actual risk of malignancy may not be gender dependent. Similarly, a 2025 study investigating 411 patients with multinodular goiter, including both retrosternal (RSG) and cervical multinodular goiter (cMNG) presentations, found a higher proportion of male patients in RSG cases (34.2%) compared to cMNG (22.9%) (p = 0.014). Despite this, the overall cancer frequency was not significantly different between genders, further indicating that gender might not be a decisive factor in thyroid malignancy risk [10].

The use of L-thyroxine to prevent further enlargement of an existing simple goiter is common practice in Sudan; however, there is no documented evidence supporting its efficacy. In some communities, goiter is not perceived as a disease, and surgical intervention is considered only in cases of marked enlargement or when pressure symptoms develop [9,11]. Although relatively rare, thyroid cancer constitutes less than 1% of all human tumors. It represents the most frequent form of cancer of the endocrine glands, accounting for approximately 90% of all endocrine cancers [12].

These management practices occur against a backdrop of significant regional variations in thyroid cancer incidence. For example, while prevalence ranges from 6% in Hawaii and Iceland to less than 1.5% in Denmark and England [7], the Middle East exhibits notably higher rates, particularly among patients with multinodular goiters. One study reported a 21.7% incidence of differentiated thyroid carcinoma among 120 patients with multinodular goiters who underwent fine needle aspiration (FNA) cytology [11]. Similarly, another study found a 12.9% malignancy rate among 147 Iraqi patients with multinodular goiters, with 74.5% identified as papillary carcinomas [13]. This pattern of increased malignancy rates suggests that long-standing multinodular goiters in iodine-deficient regions are at elevated risk of malignant transformation. The current study further supports this association, reporting a 34% incidence of thyroid cancer in long-standing goiters substantially higher than both local and global averages [7,14].

The global increase in the incidence of thyroid carcinoma has been partially attributed to exposure to ionizing radiation and the improved sensitivity of diagnostic tools, which facilitate the detection of even subclinical cases [15]. In the United States, this rising trend has sparked debate, with some researchers suggesting that the observed "epidemic" reflects enhanced diagnostic practices rather than a true surge in disease prevalence [16]. In the present study, the rate of incidental thyroid carcinoma was 54 cases (32.5%), which is notably higher than the 2.1% reported in a previous study [17]. Another study proposed that nodules within MNG lacking suspicious characteristics may be managed with routine ultrasound monitoring rather than immediate biopsy [18]. However, determining the optimal frequency and timing for these follow-ups remains challenging. Consideration must also be given to the financial costs and psychological burden associated with repeated surveillance, especially given the unpredictability of malignant transformation.

Papillary carcinomas are widely reported as the most prevalent subtype associated with multinodular goiters, representing between 60% and 74% of all thyroid malignancies [13,17,19]. Contrary to this global trend, our findings demonstrated a predominance of the follicular subtype among the study cohort, as illustrated in Table 2, where 23 patients (42.6%) were diagnosed with follicular carcinoma. Moreover, 11 patients (6.9%) with malignant goiters presented with distant metastases. This contrasts with the findings in a large cohort of 152,979 patients, which reported a distant metastasis rate of only 1.22%, with metastasis risk varying significantly by pathology and tumor size [19]. The higher rate observed in our study may indicate delayed diagnosis or more aggressive tumor behavior in long-standing goiters.

This study’s strengths lie in its comprehensive analysis of a relatively large cohort of patients with long-standing goiters in a region where data are scarce. The use of total population sampling minimized selection bias and ensured the inclusion of all eligible cases, enhancing the representativeness of the findings. Moreover, the integration of detailed clinical, biochemical, and histopathological data allowed for a thorough evaluation of malignancy risk and subtype distribution, providing valuable insights into thyroid cancer patterns in an iodine-deficient population.

However, the results should be interpreted with caution due to several limitations. The retrospective design inherently carries risks of selection and information bias, relying on the accuracy and completeness of medical records. Conducted at a single tertiary care center, the study may not fully represent the broader Sudanese population or other healthcare settings. Important confounding factors such as iodine and iron status, socioeconomic background, and healthcare access were unavailable, potentially influencing the findings. The study period (March 2017 to September 2018) was chosen based on data accessibility, especially considering subsequent conflict-related disruptions that limited more recent data collection. Additionally, the absence of a national cancer registry in Sudan restricts comparisons with nationwide incidence rates.

Despite these limitations, this study contributes valuable epidemiological data on the risk and patterns of malignancy in long-standing goiters within a resource-limited, iodine-deficient region. The observed predominance of follicular carcinoma and higher-than-expected distant metastasis rates underscore the need for early detection and structured follow-up protocols in similar settings. Future research should prioritize multicenter, prospective studies incorporating broader epidemiological variables to refine understanding and improve thyroid disease management in endemic areas.

## Conclusions

This study found a considerable rate of malignancy among patients with long-standing goiters who have undergone thyroidectomy at Khartoum Teaching Hospital, with follicular carcinoma emerging as the most common subtype. A significant number of these malignancies were incidentally discovered during histopathological examination. These findings underline the importance of thorough preoperative evaluation and patient counseling, particularly given the elevated cancer risk in long-standing goiters. Further research is recommended to explore the predictive factors for malignancy in long-standing goiters and to evaluate the effectiveness of routine FNA in early detection, especially in endemic regions.
